# Perception of acoustically complex phonological features in vowels is reflected in the induced brain-magnetic activity

**DOI:** 10.1186/1744-9081-3-26

**Published:** 2007-06-01

**Authors:** Carsten Eulitz, Jonas Obleser

**Affiliations:** 1 Department of Linguistics, University of Konstanz, Germany; 2 Max Planck Institute for Human Cognitive and Brain Sciences, Germany

## Abstract

A central issue in speech recognition is which basic units of speech are extracted by the auditory system and used for lexical access. One suggestion is that complex acoustic-phonetic information is mapped onto abstract phonological representations of speech and that a finite set of phonological features is used to guide speech perception. Previous studies analyzing the N1m component of the auditory evoked field have shown that this holds for the acoustically simple feature place of articulation. Brain magnetic correlates indexing the extraction of acoustically more complex features, such as lip rounding (ROUND) in vowels, have not been unraveled yet. The present study uses magnetoencephalography (MEG) to describe the spatial-temporal neural dynamics underlying the extraction of phonological features. We examined the induced electromagnetic brain response to German vowels and found the event-related desynchronization in the upper beta-band to be prolonged for those vowels that exhibit the lip rounding feature (ROUND). It was the presence of that feature rather than circumscribed single acoustic parameters, such as their formant frequencies, which explained the differences between the experimental conditions. We conclude that the prolonged event-related desynchronization in the upper beta-band correlates with the computational effort for the extraction of acoustically complex phonological features from the speech signal. The results provide an additional biomagnetic parameter to study mechanisms of speech perception.

## Background

It is still unresolved which are the basic units of speech that are extracted by the auditory system and used for lexical access (see [1-3] for an overview of models of speech comprehension). To address this problem in the initial steps of speech processing, the concept of distinctive features has been proposed many years ago by Jakobson, Fant & Halle [4]. That is, a set of abstract, phonological features allows the identification of acoustically quite variable exemplars of classes of speech sounds. These distinctive or phonological features can be described in speech production by means of articulatory properties [5] and in speech perception by using various acoustic/phonetic parameters in speech sounds [6,7]. From a linguistic point of view, such a set of 12 to 14 abstract and hierarchically organized features seems to be sufficient to explain the robustness and efficiency of speech recognition under various conditions like different speakers, contexts or different levels of environmental noise [8]. However, whether the human brain uses a similar approach in speech recognition is still a matter of debate. Supporting evidence comes from studies of phonological feature discrimination, e.g. voice-onset time differences [9]; which has neurophysiological support from monkey studies; [10], or differences in the place of articulation in vowels [11-13]. In the latter study, it has been shown that around 100 ms post stimulus onset (i.e., in the N100m component of the auditory evoked magnetic field) slightly different patches in the auditory cortex were activated during the extraction of the mutually exclusive place of articulation-features dorsal and coronal. However, other phonological features in which the vowels differed as well like lip rounding (ROUND) did not systematically influence the N100m.

The present study reanalyzes the raw data of the Obleser et al. [12] study with the aim to describe further parameters indexing the extraction of other phonological features. For this purpose and in contrast to the original strategy of data analysis, we studied the so-called induced brain activity, which is time- but not phase-locked to the event-related brain activity (for review see [14-16]. This data analysis technique visualizes the non-phase locked brain activity which is largely averaged out when using conventional averaging techniques such as event-related potentials or fields. In sum, the evoked and the induced brain activity are delivering complementary information about perceptual processes. The rationale for the alternative way of data analysis is the following: The more complex the stimuli and the more acoustic variance is in the stimuli belonging to one particular experimental condition and the higher the level of information processing under investigation, the higher we expect the variance of the timing of a particular cognitive process to be. In the present study with six tokens for each of the seven spoken German vowels, it is reasonable to assume such variability, especially for abstract phonological features where several acoustic low-level features have to be integrated.

**Figure 1:**
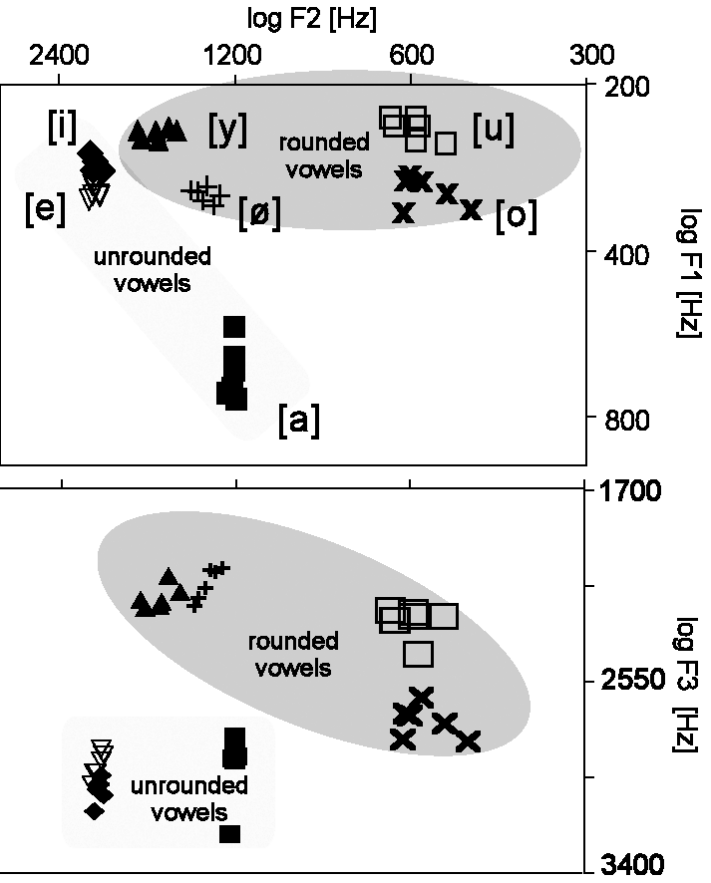
Upper panel: A vowel space plotting first formant frequency (y axis, logarithmic display) against second formant frequency (x axis, logarithmic display) is shown for all vowel exemplars used. Lower panel: Third formant frequency (y axis, logarithmic display) against second formant frequency (x axis, logarithmic display) is shown. Note the considerable acoustic variance within vowel categories, and that no single formant dimension alone predicts the roundedness.

Different phonological features seem to differ with respect to the required amount of acoustic feature integration. For instance, tongue height can presumably be defined on the basis of a cut-off criterion for the first formants' frequency. Other phonological features, like ROUND, are more difficult to handle. It is widely accepted that the third formant's frequency is important for perceiving ROUND, but this formant alone is not enough for the perfect detection of ROUND in speech signals (for illustration see also Fig. 1). To our knowledge, no acoustic/phonetic parameter has been identified allowing a near-perfect detection of ROUND in speech. Recent automatic speech recognition systems conceptually based on using phonological features [8] are practically blind for ROUND, because it cannot be described as a linear combination of well defined acoustic/phonetic parameters. Consequently, ROUND is a candidate feature that requires higher level information processing, effects of which might be averaged out when using phase-locked averaging techniques. The analysis of the induced brain activity might therefore unravel processes related to the extraction of this complex feature.

**Figure 2:**
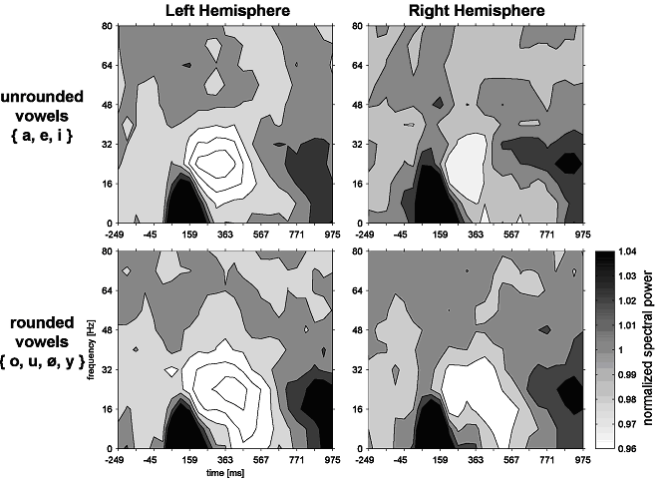
Time-frequency plots for the left and the right hemisphere (left and right column; spectral power was collapsed across 34 channels over each hemisphere) are shown separately for unrounded vowels (upper panels) and rounded vowels (lower panel). The grayscale intensity codes the standardized change of power in the respective frequency band compared to baseline. Note that the relative suppression in the 16–32 Hz band is sustained longer in rounded vowels, and that the relative changes are more pronounced over the left hemisphere.

When analyzing the induced brain activity, the outcome can be a reduction or an enhancement of the spectral power relative to the baseline interval in defined time-frequency bins. An enhancement is usually interpreted as an event-related synchronization of neural activity which might index feature binding processes [14,17-19], whereas an reduction in spectral power is usually interpreted as an event-related desynchronization (ERD) of neural activity. Based on the observation that increased cellular excitability in thalamo-cortical networks leads to desynchronization in the EEG [20], it has been proposed that the ERD is correlated with the amount of cortical activity [15], i.e. the more information processing is going on the larger is the ERD. Factors like task complexity, attention or mental effort modulated the ERD in the expected way (see [15] for review). The interpretation of ERD is depending on the frequency band, where alpha ERD is usually interpreted as increased cortical activation and gamma ERD as an index for cortical deactivation. A recent overview over changes in the induced brain activity during language processing is given by Bastiaansen and Hagoort [21].

Based on these considerations, we analyzed the induced brain responses during active listening to natural speech sounds and detecting target vowels. In order to accomplish this task, extraction and categorization of all crucial features is inevitable. Consecutively, we would expect for the present study a more intense ERD when the extraction of acoustically complex features such as round is required as compared to the processing of unrounded speech sounds. A more intense ERD can be reflected in a larger amplitude and/or a longer duration of the ERD [22-24].

## Methods

The subjects, stimulus material, experimental design and data collection was reported in detail in [12]. From the analysis of the MEG raw data on, the present study differs from the previous reported one. We will first shortly summarize the basic methodological information and then describe the analyses of the induced brain activity.

### Subjects

Data of 13 subjects (seven females) with a mean age of 24.8 ± 4.6 years (M ± SD) were analyzed. As a good signal to noise ratio is crucial for time-frequency analyses, we used only those subjects which formerly passed the criteria for the source analyses. Unfortunately, one subject of those could not be analyzed, because of technical problems in de-archiving the raw data in one of the recording blocks. None of the participants reported a history of neurological, psychiatric, or otological illness. All subjects were monolingual native speakers of German. Only right-handers were included, as ascertained by a right-handedness score > 90 in the Edinburgh Handedness Questionnaire [25]. Subjects gave written informed consent and were paid € 20 for their participation.

### Stimulus material and experimental design

We investigated brain responses to seven naturally spoken German vowels. Some of them were unrounded, [a], [e], [i] (as in "father", "bay", "bee", respectively) and others were rounded, like [o], [u] (as in "doe" and "do"), or [ø], [y] (as in "Goethe" and "Dürer"). The latter two vowels are the rounded counterparts of the front vowels [e] and [i], and do not occur in English. The classification of the vowels in terms of their phonological feature ROUND as well as their pitches and formant frequencies are given in Table 1 and Figure 1. For every vowel category, we selected six tokens resulting in 42 different stimuli. Vowels were cut out of words spoken by a male speaker. To prepare the stimuli, lists of words (several for each vowel) were articulated and recorded with artificially long lasting vowels (~300 ms), so that segments free of any coarticulation could be extracted. In a pilot rating study, the 10 best for each vowel category with a typical variation of fundamental and formant frequencies was selected. From the 10 kHz-digitized speech signal, 350 ms portions containing only the steady-state vowel were used. Stimuli were first equalized for the root mean square (rms), than normalized for peak amplitude and finally ramped with 50 ms Gaussian on- and offsets. Pitch frequency (119 ± 10 Hz, M ± SD) and formant frequencies varied within vowel categories (cf. Fig. 1 and Table 1), thus introducing considerable acoustic diversity.

**Table 1:** Overview over the assignment of the phonological feature ROUND as well as the pitch and formant frequency variability in the vowel categories used.

**Vowel category**	**ROUND**	**F**_**0 **_**min-max**	**F**_**1 **_**min-max**	**F**_**2 **_**min-max**	**F**_**3 **_**min-max**
**[a]**	**-**	103–113	552–747	1188–1224	2663–3171
**[i]**	**-**	127–132	267–287	2048–2120	2838–3028
**[e]**	**-**	109–125	302–322	2055–2143	2711–2890
**[y]**	**+**	115–144	238–248	1516–1769	1978–2097
**[ø]**	**+**	108–125	301–325	1293–1447	1945–2079
**[u]**	**+**	112–118	231–256	522–645	2117–2292
**[o]**	**+**	109–125	293–346	471–609	2481–2688

F0 refers to the pitch and F1, F2, F3 to the corresponding formant frequencies.

Vowels were presented binaurally with 50 dB SL via a non-magnetic echo-free stimulus delivery system with almost linear frequency characteristic in the critical range of 0.2–4 kHz. Vowels were aligned in pseudo-randomized sequences of 572 stimuli with a stimulus onset asynchrony ranging randomly between 1.6 and 2.0 s. Every subject listened to three of such sequences. To sustain subjects' attention to the stimuli, a target detection task was employed: In every sequence, two given vowels had a low probability of occurrence (10% together) and served as targets. Subjects had to press a button with their right index finger when they detected a target. As all vowel categories exhibited acoustic variance, subjects had to map stimuli onto vowel categories to decide whether a given stimulus is a target or not, i.e. subjects had to maintain a phonological processing mode throughout the experiment. The reasons to use 2 vowels as targets in one block and different vowels across blocks are outlined below:

(1) We wanted to have at best all vowels serving as possible targets across the whole experiment to avoid that a certain anchor point in the vowel space would transform the perceptual space.

(2) We wanted to have more than one vowel as a target in one block of measurement (i) to reduce the possibility to carry out the task by a simplistic auditory pattern matching strategy and thus making the phonetic processing mode less salient; (ii) to avoid that a strategy of attending to just one featural dimension in one block of measurement (i.e. tongue height or place of articulation) would be sufficient to solve the task – this was important to reduce the possible confound of attention to certain feature dimensions. (3) We wanted to have all target vowels with the same probability being a target.

Given those constraints and the odd number of vowels in the study (to cover a substantial part of the German vowel space and having a parametric variation along the featural dimensions) we had to accept to avoid [ø] being a target. We hoped that the resulting complex target definition procedure will hide for the subjects the fact that [ø] had a relative probability of only 98.3% and was never one of the two targets across the multiple blocks of measurement. Indeed, none of the subjects reported after the experiment, that [ø] was never a target

Prior to the recordings, subjects repeated vowel stimuli aloud and recognized all stimuli as typical German vowels. Binaural loudness was slightly readjusted to ensure the auditory perception in the midline. Subjects watched silent videos in order to maintain constant alertness and to reduce excessive eye movements.

### Data acquisition and reduction

Neuromagnetic fields were recorded using a whole head system (MAGNES 2500,4D Neuroimaging, San Diego) in a magnetically shielded room (Vacuumschmelze, Hanau, Germany). The measuring surface of the sensor is helmet shaped and covers the entire cranium. Within the sensor, 148 magnetometer signal detectors are arranged in a uniformly distributed array spaced by 28 mm. Subjects were measured in lying position. They were instructed to avoid eye blinks and head movements, and to carry out the monitoring task carefully. Continuous data were recorded in 20-minute blocks at a sampling rate of508.6 Hz within a pass band of 0.1 to 100 Hz.

For analyses of the induced brain activity, a method similar to event-related perturbation analyses [26] was applied using the avg_q software [27]. In artifact-free epochs of MEG raw data (signal deviations of more than 3.5 pT in the MEG or erroneous button presses on non-target vowels as well as target vowels were excluded), power spectral estimates were derived from Fourier transforms on pairs (overlapping by one half) of 188.75 ms Welch-windowed segments. Under these constraints, a frequency resolution of 7.94 Hz was obtained. Power estimates were selectively averaged for each segment around stimulus onset and stimulus class. Nine time segments were situated equidistantly within a 600-ms interval before stimulus onset (baseline), and 21 segments after stimulus onset within the total interval of 1700 ms. The mean power spectra were transformed with respect to baseline-related changes. Normalized mean power spectra were calculated by dividing each single mean spectral power estimate within one time-frequency bin by the mean spectral power estimate across all corresponding baseline segments.

**Figure 3:**
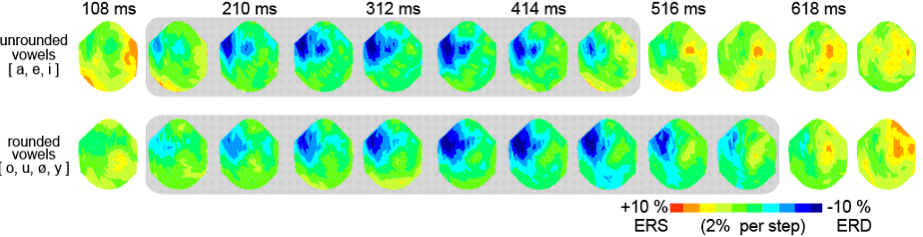
Top views (projection onto a plane; nose is on top and right side on the right) of 16–32 Hz band topographies from ~100 to 650 ms post stimulus onset are shown for unrounded (upper row) and rounded vowels (lower row). The event-related desynchronization (ERD, blue) is markedly sustained in rounded vowels and most prominent over left anterior sites (scaling 110 - 90% relative power change, color steps indicating 2% change; ERS, event-related synchronization). The grey background highlights the time range with the most pronounced ERD.

**Figure 4:**
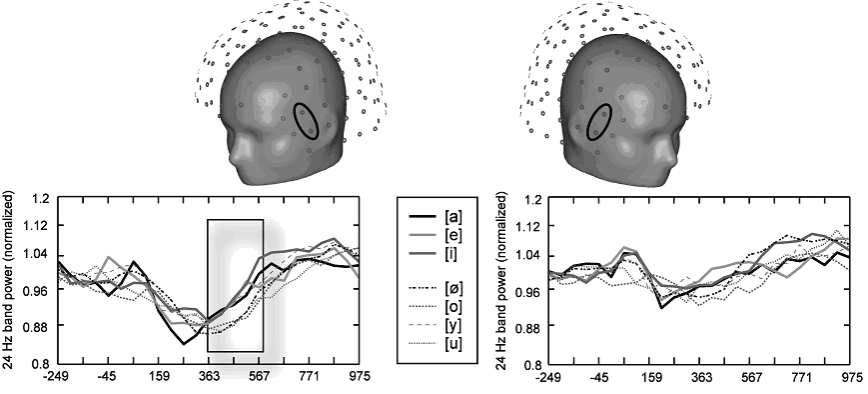
Time courses of relative changes in 24 Hz band power from left- and right-hemispheric anterior sites are displayed for all seven vowel categories. As best seen in the highlighted box, unrounded vowels (thick lines) do not show the sustained suppression as it is evident in rounded vowels (dashed lines). Head shapes in the upper panels illustrate the location of the displayed channels in the sensor array.

To further reduce the data and to obtain the most relevant time/frequency bins based on estimates that are not affected by small alterations in the location or orientation of the generating brain regions, and that show little dependency on individual variations of brain anatomy, the normalized spectral power was collapsed across 34 recording channels centered over the left and 34 over the right hemisphere, respectively [12]. For the time/frequency bin of interest (which was in the upper beta-band for the present study), 6 pairs of channels located along an anterior-posterior line capturing the maximum spectral power changes were used for more detailed topographical analyses (see also Fig. 3 &4). In order to account for small alterations in the individual topography and to enhance the signal-to-noise ratio, the inferior and superior recording channel of each pair were collapsed (see upper panels in Fig. 4 for illustration).

### Statistical analysis

Data from the upper beta-band (frequency band around 24 Hz, which showed the maximum induced spectral power changes in higher frequency bands across all experimental conditions) at two different time bins (covering the maximum induced spectral power changes) were initially compared in a 2 × 2 × 7 repeated measures analysis of variance with factors TIME BIN (450 ms, 550 ms), HEMISPHERE (left channels, right channels) and VOWEL CATEGORY (all seven vowels). The first time bin was chosen in a way to cover the maximum ERD across all channels and subjects in all conditions. The second time bin was then shifted 100 ms to reduce the overlap of time windows for the spectral power estimate while still covering the cognitive process of interest. An amplitude difference between the experimental conditions in the second but not the first time window (as reflected in an interaction) would index a prolonged cognitive process of interest in a subset of conditions.

A specific test for the influence of the ROUND was then performed in a reduced 2 × 2 × 2 design with factors time bin (450 ms, 550 ms), hemisphere (left channel group, right channel group) and round (mean of unrounded vowels [a, e, i] vs. mean of rounded vowels [o, u, ø, y]).

Additionally, a topographical change over time was tested by introducing an additional six-fold factor topography (six pairs of channels ranging from posterior-temporal to anterior-temporal sites).

We also tested whether the selective beta-band desynchronization was systematically different from the N100m-related topography. Therefore, the ERD topography, as represented by the ERD change along the anterior-posterior line was compared with the enhancement of normalized spectral power in the time/frequency bin centered around 100 ms and 8 Hz in the same pairs of channels. For comparability, we standardized the percentage of power change in both bands according to McCarthy and Wood's transformation [28] and then tested a 2 × 2 × 6 × 7 repeated measures analysis of variance with factors time-frequency bin (24 Hz around 450 ms, 8 Hz around 100 ms), hemisphere (left channels, right channels), topography (six pairs of channels ranging from posterior-temporal to anterior-temporal sites) and vowel category (all seven vowels). Where necessary (i.e. where a violation of the homogeneity of variances assumption was evident by Mauchly's criterion), Greenhouse-Geisser-adjusted p-values are reported.

## Results

As Figure 2 shows, the event-related desynchronization (ERD) changed as a function of time bin [F(1,12) = 7.0, p < .03], in that the beta-band power regained amplitude in the 550 ms time bin. However, not all vowel categories behaved similarly over time [vowel category × time bin interaction; F(6,72) = 2.3, p < .05]. This permitted a more specific testing of the influence of ROUND. The time bin × round interaction attained significance [F(1,12) = 11.3, p < .01]: It was only in rounded vowels that the ERD was sustained (Figs. 2, 3; lower panels) across both time bins (normalized 24 Hz band power 0.949 at 450 ms; 0.950 at 550 ms), whereas no ERD in the later time bin was present for unrounded vowels (0.947 at 450 ms; 0.981 at 550 ms).

As demonstrated in Figure 3, the ROUND-related ERD was most pronounced over left anterior sites. To further specify the topographical aspects of the ERD, the power change over hemispheres, time bins and round was tested by introducing the additional repeated measures factor topography (6 pairs of channels ranging from posterior-temporal to anterior-temporal sites). This analysis yielded a ROUND × topography interaction [F(5,60) = 5.6, p < .001]: The ERD topography gradient with stronger ERD over more anterior sites was manifest in rounded vowels [F(5,60) = 6.6, ε = .34, p < .01] but not in unrounded vowels [F(5,60) = 1.7, ε = .38, p > .20].

Left-hemispheric ERD effects were generally stronger [F(1,12) = 38.0, p < .0001], but a topography × hemisphere interaction was also evident [F(5,60) = 13.2, ε = .42, p < .0001]: channel sites in the right hemisphere showed no topographic effects whatsoever (topography effect n.s.; cf. Fig. 3). Figure 4 shows the power change in the 24 Hz band over time for the same pair of channels over the left and the right hemisphere and illustrates the hemispheric asymmetry of this round-related ERD process.

To ensure that the selective beta-band ERD was systematically different from the topography of the N1m-P2m complex which exhibits the highest spectral power in the 5–15 Hz range, the ERD power in the 24 Hz/400 ms-centered time-frequency bin was compared to the enhancement of normalized spectral power in the time-frequency bin centered around 100 ms and 8 Hz across the same pairs of channels. A topography × time-frequency bin interaction proved that there were topographical differences between the N100m-related activity and the ERD response [F(5,60) = 16.1, ε = .28, p < .001]. Signal change was also largest over anterior-temporal sites in the 100 ms/8 Hz bin, but exhibited a second peak in signal change over posterior-temporal sites, whereas the ERD response showed only the described anterior-temporal peak in the left hemisphere (Figs. 3, 4).

## Discussion

The present study showed a prolonged event-related desynchronization in the upper beta-band of the induced brain activity whenever the phonological feature ROUND was present in naturally spoken vowels. Due to the complex distribution of the acoustic features in the speech sounds, this effect can be best explained by the presence of that feature rather than circumscribed single acoustic parameters, such as their formant frequencies (Fig. 1). The topography of this effect is different from that induced by the N1m-P2m-complex which is reflected as an enhanced spectral power in the alpha-band. The topographical difference suggests that the ERD in the upper beta-band is generated in different brain areas. The prolonged ERD in the upper beta-band during the extraction of the feature ROUND was evident for the general comparison amongst all vowels (cf. Fig. 4).

Event-related desynchronization has been proposed to be correlated with the amount of cortical activity, and factors like task complexity, attention or mental effort do modulate the ERD [15]. In general, enhanced mental effort for various reasons leads to a more pronounced ERD. Thus, the prolonged ERD in the upper beta-band during the extraction of the feature ROUND as found in the present study can be interpreted most suitably as an index of the enhanced computational effort for the extraction of an acoustically complex phonological feature. Interestingly, in automatic speech recognition, researchers are faced with a similar problem and to our knowledge phoneticians have no reliable algorithm for the detection of ROUND in speech. More simple phonological features, such as place of articulation and tongue height are highly correlated with changes in formant frequencies F1 and F2 [29] and can accordingly be handled by automatic speech recognition [8]. Accordingly, those features were also investigated more successfully in neuroimaging studies [12,13,30,31]. However, lip rounding appears to require more complex operations along the auditory pathway resulting in more subtle effects requiring alternative methods of data analyses. Analysis of the induced brain activity seems to be a feasible tool to study these processes.

The study supports the notion of beta-band ERD as a correlate of mental effort and provides an additional biomagnetic parameter for studies of speech perception. This is to our knowledge the first study reporting a brain signature for the detection of ROUND in speech: all parameters reported so far including those of our own analyses of the evoked brain activity in the same set of raw data [12] were not sensitive to the phonological feature ROUND.

Intriguingly, a previous ERD mapping study reported an enhanced beta-band suppression in response to words over fronto-temporal electrodes roughly similar to the anterior channels showing most vigorous responses in present MEG data [24]. Roughly the same brain region has been involved in tasks requiring auditory working memory [32,33]. In this task, Kaiser and colleagues reported, however, enhanced gamma-band synchronization. In sum, the conclusion that beta-band ERD mirrors enhanced processing load appears justified for neural networks dedicated to the auditory input-to-meaning mapping.

The effect of lip rounding we see here occurs comparably late, especially so in comparison to the place of articulation feature which has been suggested to affect the N100m [12] and to a certain extent even the P50m [34] component of the evoked field. Why should a more subtle vowel feature change exhibit its influence much later in the speech sound decoding process? Besides any potential temporally blurring effects of the comparably broad Welsh window chosen for increased accuracy in frequency estimation (189 ms), it is worth remembering that listening to isolated vowels in order to categorize them and to react accordingly might involve a cascade of evaluation and re-evaluation processes while disambiguating the vowel percept. These processes might or might not be necessary in real life speech recognition (depending on whether or not such a minute feature is needed to disambiguate a lexical item), and this should be put to test using minimal word pairs rather than isolated vowels. In general, however, it seems fruitful to distinguish speech *perception *tasks as the one employed here (which require attention to subphonemic detail and may strongly involve phonological working memory and other supporting cognitive processes depending on the nature of the task, see [3] for further reference) from speech *recognition *tasks which inevitably involve access to the mental lexicon. Hence, bringing these levels of speech processing together and utilizing subtle featural differences such as lip rounding in experiments that do require natural speech recognition will help to disambiguate the role of the beta ERD seen here.

This experiment cannot provide automatic speech recognition systems with an algorithm for the detection of ROUND in speech, but it has become clear that such an algorithm will have to be more complex than those for the detection of the other phonological features in the vowels. Since telling an [y] from an [i] is nevertheless accomplished with great ease by all speakers of languages with umlauted vowels, the results reported here are encouraging to strive for new and more derivative parameters reflecting the speech perception process: Understanding the brain's effortless decoding of these acoustically more complex features in speech will ultimately lead to a more general model of the human speech perception faculty.
